# Using Systems Engineering and Implementation Science to Design an Implementation Package for Preoperative Comprehensive Geriatric Assessment Among Older Adults Having Major Abdominal Surgery: Protocol for a 3-Phase Study

**DOI:** 10.2196/59428

**Published:** 2024-09-09

**Authors:** Julia R Berian, Margaret L Schwarze, Nicole E Werner, Jane E Mahoney, Manish N Shah

**Affiliations:** 1 Department of Surgery School of Medicine and Public Health University of Wisconsin-Madison Madison, WI United States; 2 Division of Geriatrics and Gerontology, Department of Medicine School of Medicine and Public Health University of Wisconsin-Madison Madison, WI United States; 3 Department of Health and Wellness Design School of Public Health Indiana University-Bloomington Bloomington, IN United States; 4 BerbeeWalsh Department of Emergency Medicine School of Medicine and Public Health University of Wisconsin-Madison Madison, WI United States

**Keywords:** systems engineering, participatory design, user-centered design, implementation science, surgery, aging research, randomized controlled trial

## Abstract

**Background:**

Older Americans, a growing segment of the population, have an increasing need for surgical services, and they experience a disproportionate burden of postoperative complications compared to their younger counterparts. A preoperative comprehensive geriatric assessment (pCGA) is recommended to reduce risk and improve surgical care delivery for this population, which has been identified as vulnerable. The pCGA optimizes multiple chronic conditions and factors commonly overlooked in routine preoperative planning, including physical function, polypharmacy, nutrition, cognition, mental health, and social and environmental support. The pCGA has been shown to decrease postoperative morbidity, mortality, and length of stay in a variety of surgical specialties. Although national guidelines recommend the use of the pCGA, a paucity of strategic guidance for implementation limits its uptake to a few academic medical centers. By applying implementation science and human factors engineering methods, this study will provide the necessary evidence to optimize the implementation of the pCGA in a variety of health care settings.

**Objective:**

The purpose of this paper is to describe the study protocol to design an adaptable, user-centered pCGA implementation package for use among older adults before major abdominal surgery.

**Methods:**

This protocol uses systems engineering methods to develop, tailor, and pilot-test a user-centered pCGA implementation package, which can be adapted to community-based hospitals in preparation for a multisite implementation trial. The protocol is based upon the National Institutes of Health Stage Model for Behavioral Intervention Development and aligns with the goal to develop behavioral interventions with an eye to real-world implementation. In phase 1, we will use observation and interviews to map the pCGA process and identify system-based barriers and facilitators to its use among older adults undergoing major abdominal surgery. In phase 2, we will apply user-centered design methods, engaging health care providers, patients, and caregivers to co-design a pCGA implementation package. This package will be applicable to a diverse population of older patients undergoing major abdominal surgery at a large academic hospital and an affiliate community site. In phase 3, we will pilot-test and refine the pCGA implementation package in preparation for a future randomized controlled implementation-effectiveness trial. We anticipate that this study will take approximately 60 months (April 2023-March 2028).

**Results:**

This study protocol will generate (1) a detailed process map of the pCGA; (2) an adaptable, user-centered pCGA implementation package ready for feasibility testing in a pilot trial; and (3) preliminary pilot data on the implementation and effectiveness of the package. We anticipate that these data will serve as the basis for future multisite hybrid implementation-effectiveness clinical trials of the pCGA in older adults undergoing major abdominal surgery.

**Conclusions:**

The expected results of this study will contribute to improving perioperative care processes for older adults before major abdominal surgery.

**International Registered Report Identifier (IRRID):**

DERR1-10.2196/59428

## Introduction

### Background

Each year, approximately 4.5 million older adults undergo major surgery [1]. On average, 25% of these older adults develop postoperative complications, costing the health care system approximately US $8000 to US $13,000 per complication [2] and an average of US $20,000 to US $30,000 for the postoperative hospitalization [3-6]. Unfortunately, the risk of complications doubles with preoperative functional decline, frailty, or cognitive impairment [7-14], which are risk factors that are rarely measured. A preoperative comprehensive geriatric assessment (pCGA) is a multicomponent intervention evaluating and optimizing multiple chronic conditions and factors commonly overlooked in routine preoperative planning, including physical function, polypharmacy, nutrition, cognition, mental health, and social and environmental support [15]. Major abdominal surgery carries unique risks, disrupting the core muscles with often-delayed functional recovery [16-21], cognitive decline [17,20,22-24], and disproportionately high morbidity and mortality [7,25-28]. When implemented properly, the pCGA improves outcomes for patients undergoing abdominal surgery, reducing postoperative complications by 40% and length of stay by 2 days [29,30].

Experts and best practice guidelines recommend a pCGA for all older patients undergoing major surgery [15,31-33]. In prior work with the American College of Surgeons, an expert consensus conference of >50 stakeholder organizations generated national standards for the surgical care of older adults through the Geriatric Surgery Verification Program [34,35]. These standards include conducting a pCGA for older surgical patients. Defining the standards is a critical first step; however, this work is incomplete without generalizable implementation strategies applicable to real-world settings. A lack of evidence on how best to implement the pCGA in a variety of health systems has limited patient access to this intervention [36]. The few implementation studies that exist demonstrate challenges due to contextual factors (eg, misaligned incentives) and low fidelity to the intervention [36,37]. As a result, few programs use the pCGA for older surgical patients, and reach is limited to patients receiving care at large academic medical centers with significant resources. Thus, there is a critical need for novel, evidence-based strategies to promote the successful implementation of this intervention in a variety of health care settings. To address this gap, this study seeks to answer the research question “What implementation strategies will improve provider use (adoption) and patient access (reach) to the pCGA, while preserving effectiveness (fidelity) in academic and community medical settings?”

### Objectives

The overall objective of this study is to use implementation science and systems engineering methods to tailor and pilot-test a user-centered pCGA implementation package that can be adapted to community-based hospitals for future multisite implementation-effectiveness trials. Therefore, we aim to (1) map the pCGA process and identify system-based barriers and facilitators to its use among older adults undergoing major abdominal surgery, (2) co-design a pCGA implementation package applicable to a diverse population of older patients undergoing major abdominal surgery at a large academic hospital and an affiliate community site, and (3) test and refine the pCGA implementation package in preparation for a future randomized controlled implementation-effectiveness trial. We hypothesize that systems engineering methods of process mapping and co-design can successfully be applied to the pCGA (aims 1 and 2) and that a rigorous user-centered pCGA implementation package will improve surgical care processes (reach and adoption, which are the primary implementation outcomes) and patient outcomes (length of stay, which is the primary effectiveness outcome) for older adults undergoing major abdominal surgery.

## Methods

### Design and Conceptual Framework

This study will apply implementation science and human factors engineering principles to optimize the delivery of an existing, effective intervention—the pCGA—and generate an adaptable implementation package for future work. Through process mapping, supplemented with observation and interviews, we will identify opportunities for system redesign that will improve pCGA delivery (phase 1) and use participatory design with patients, caregivers, and health care providers to develop a user-centered implementation package (phase 2) that is adaptable to academic and community settings. Subsequently, we will pilot-test the implementation package (phase 3) using the reach, effectiveness, adoption, implementation, and maintenance (RE-AIM) framework [38], generating preliminary data in preparation for a larger randomized controlled implementation-effectiveness trial. This work aligns with the National Institutes of Health (NIH) Stage Model [39] for intervention development, considering implementation early in development (stage IA) and recognizing that the development and evaluation of complex interventions may not be linear.

The pCGA (Table 1) is based on a model by McDonald et al [29] that has demonstrated decreased length of stay, readmission rates, and discharge to nonhome destination for patients undergoing abdominal surgery. To improve outcomes, the pCGA must be implemented with fidelity to key elements [15]. Treatment fidelity, particularly for complex behavioral interventions, is conceptualized as consistency in the content of the intervention, the quality of intervention delivery by health care professionals, and the receipt and adherence by patients [40]. The content of the pCGA requires a multidomain assessment [41,42]; however, equally important are the presence and quality of a follow-up plan to optimize identified deficits, as well as patient adherence to this plan. The pCGA often generates recommended tasks for the patient (exercises and nutritional shakes) and provider (medication changes) or identifies additional preoperative workup to be performed (imaging or physiological testing). Many studies on pCGA fall short by using it primarily as a risk stratification tool without a plan or sufficient time to address modifiable factors [43-45]. In addition to the aforementioned study by McDonald et al [29], studies from the United Kingdom have demonstrated that the pCGA, performed in multidisciplinary settings with a plan to address deficits, reduces postoperative length of stay and complications [30,46]. These studies have led to national and international guidelines that recommend a pCGA for older adults before major surgery [15,31-33].

**Figure 1:**
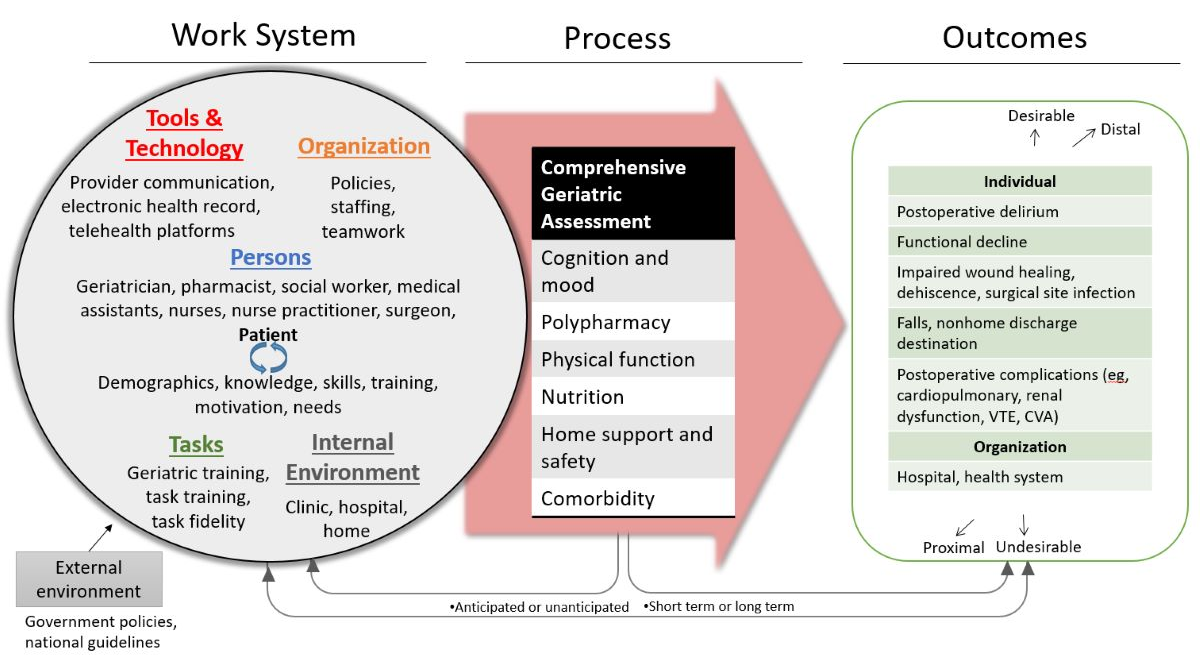
Preliminary systems engineering initiative for patient safety model 2.0 depicting the preoperative comprehensive geriatric assessment process. CVA: cerebrovascular accident; VTE: venous thromboembolism.

Systems engineering methods improve health care delivery and outcomes by tailoring the fit of the intervention to the local context. A seminal report from the Institute of Medicine and the National Academy of Engineering outlines the unique methods and tools by which systems engineering can transform the quality and delivery of health care [47]. We will use a human factors engineering model: the systems engineering initiative for patient safety (SEIPS) model [48]. It builds on the structure-process-outcome model developed by Donabedian [49] by providing a detailed and expanded structure of the interacting components (ie, the work system)—people (or teams), tasks, tools and technology, organizational factors (eg, policies and teamwork), physical environment (clinic location), and external environment—that influence health care processes and outcomes. SEIPS has been extensively and successfully applied to health care delivery [50-58], including work to improve care transitions for older adults [59-64]. We will use SEIPS 2.0 [65], which expanded the original model [48] to acknowledge that patients and families perform work within the health care process and that their internal environment (ie, patient home) plays a role within the system (Figure 1).

**Table 1:** Preoperative comprehensive geriatric assessment.

Domain	Assessment	Staff performing the assessment	Example output
Polypharmacy	Medication reconciliation Identification of high-risk Beers criteria medications Anticholinergic cognitive burden score	Pharmacist	Medication changes made directly or referred to PCP^a^
Medical comorbidities	History and physical examination, including cardiac risk assessment with revised cardiac risk index	Geriatrician	Additional testing ordered; specialist referral placed
Physical function	Mobility testing (eg, Timed Up and Go Test) Lawton Independent Activities of Daily Living scale, activities of daily living, and home support and resources	Medical assistant and social worker	Home exercises prescribed or preoperative physical therapy referral (“prehabilitation”)
Cognition	Delirium risk assessment (eg, Montreal Cognitive Assessment)	Social worker	Memory clinic referral
Mood	Depression screen (eg, Patient Health Questionnaire-9)	Medical assistant	Medication or specialist referral
Nutrition	Nutritional status (eg, Mini Nutritional Assessment–Short Form) and BMI	Medical assistant	Nutritional supplementation
Goals of care	Discussion and documentation of acceptable outcomes and code status Assist patient with documenting health care power of attorney	Geriatrician	DNR^b^ status established and communicated to surgeon

^a^PCP: primary care provider.

^b^DNR: do-not-resuscitate.

The study was registered at ClinicalTrials.gov in accordance with NIH policies and regulations (phases 1 and 2 [observational phases]: NCT06184919; phase 3 [pilot trial]: NCT06184724).

### Ethical Considerations

The study will be conducted in 3 phases. Each phase will be reviewed separately by the institutional review board (IRB) of the University of Wisconsin–Madison. Phase 1 of the study was approved by the IRB on January 19, 2023 (2022-1516). All participants provide informed consent for both observations and interviews. To protect privacy and confidentiality, all data are deidentified. Compensation is provided in the form of gift cards. Participants receive US $20 for participating in observations and US $20 for a 45-minute qualitative interview. Health care staff receive US $45 for a 90-minute focus group. Surgeons receive US $50 for a 45-minute interview. Phase 2 of the study was approved by the IRB on March 1, 2024 (2024-0044). All participants provide informed consent. To protect privacy and confidentiality, all data are deidentified. Compensation is provided in the form of gift cards. Patient and caregiver participants receive US $50 per 90-minute session. Health care professionals receive US $100 per 90-minute session. Phase 3 study activities will be initiated after receiving approval from the IRB. All participants will be required to provide informed consent. To protect privacy and confidentiality, all data will be deidentified. Compensation will be provided in the form of gift cards. Participants will receive US $5 per completed survey.

### Phase 1: Process Mapping

#### Study Design

We will perform process mapping [66,67] via direct clinical observation of the pCGA visit, qualitative interviews with patients, and a focus group with staff, using SEIPS as a framework [60,67,68]. Process mapping is a fundamental systems engineering approach based upon the recognition that work, as it is performed in real-world settings, is not always consistent with its expected or prescribed form. We expect that phase 1 will catalog components of the SEIPS-based work system (traditional process map), and we will supplement the map by identifying barriers and facilitators to each step.

#### Participants

##### Patients and Caregivers

Eligible participants are patients aged ≥85 years or those aged ≥65 years with multiple comorbid conditions (>2), diagnosed or suspected cognitive impairment, diagnosed or suspected functional impairment, or poor nutrition (documented weight loss of 10% within a year). Inclusion criteria are older adults for whom the surgeon recommends an operative intervention and who intend to undergo the elective major abdominal surgery. Exclusion criteria are non-English speakers (because we do not have the resources for translation) or the absence of a caregiver for those without decisional capacity. On the basis of prior literature [68], we will enroll 20 older adults and their caregivers, if present, for observation and interviews. We will use purposive sampling to recruit at least 2 patients each for groups traditionally underrepresented in medical research by race, ethnicity, socioeconomic status, and rural or urban backgrounds [69]. Research staff will screen patients for eligibility. Clinic staff will approach participants to assess interest, and research staff will obtain informed consent.

##### Health Care Professionals and Staff

All health care professionals working in the preoperative geriatric clinic (n=10) who are responsible for performing the tasks of the pCGA will be approached and consented for direct observation and focus groups to provide feedback on the pCGA process map. Additional staff (n=5) engaged in the clinic (administrative leads and schedulers) will be invited to participate. We will invite referring surgeons (n=10) for one-on-one interviews, with the plan to recruit additional participants as informed by thematic saturation during the ongoing analysis. We will purposively select surgeons, men as well as women, to represent a range of surgical specialties. Recruitment will occur by email for health care professionals.

#### Predicted pCGA Process Map

**Figure 2:**
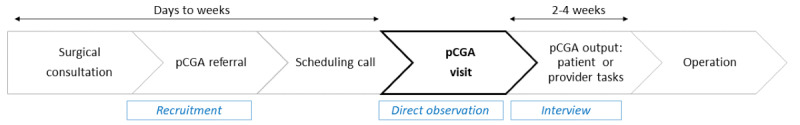
Predicted process map of patient journey through the preoperative comprehensive geriatric assessment (pCGA). Each point along the process will be evaluated using the systems engineering initiative for patient safety to identify key work tasks and associated barriers. Research activities are noted in blue italics.

The pCGA clinic is conducted after the initial surgical consultation and before the surgical operation. A predicted map of the existing process is outlined in Figure 2. The patient consults with a surgeon who recommends an operative intervention and places a referral for the pCGA clinic. The pCGA is conducted at 2 locations and provides web-based options for patients traveling from a distance. The pCGA results include recommendations for patient-level tasks (exercises and nutritional shakes), provider tasks (medication changes), or additional preoperative evaluation.

#### Data Collection

##### Direct Observation

We will embed a trained research assistant at the clinic and use direct observation guided by a fidelity checklist to document the events of the pCGA and take notes on the process. We will follow the patient from initial check-in through the completion of the visit, providing a detailed and direct account of the patient’s journey [66]. The observers will use a standardized template to document the persons, tasks, tools and technology, environment, and organizational factors according to the SEIPS model. Observer notes will be supplemented with an audio recording of the patient-physician interaction.

##### Interviews

We will interview patients and caregivers 2 weeks after the pCGA visit. Research staff trained in qualitative methods will conduct the interviews using a semistructured interview guide to elicit feedback on the predicted process map (Figure 2). The interview probes will assess barriers, facilitators, motivation, and adherence to patient work tasks, as well as reflections on the process of scheduling, attending, and participating in the pCGA. We will conduct interviews using a virtual platform. The interviewer will ask the patient to visually show on screen the home environment and ask probing questions to gain a more in-depth understanding of how tasks are performed. We expect each interview to last 45 minutes. We will interview surgeons using semistructured guides to elicit feedback on the process map and any barriers or facilitators according to the SEIPS model. The interview will provide an understanding of perceived barriers and facilitators to the adoption of the pCGA and explore how the surgeons’ separate workflow fits into the proposed process map. Surgeon interviews are expected to last 45 minutes.

##### Focus Groups

Qualitative research staff will engage pCGA health care professionals and staff (n=10) in a focus group after completing direct observations. Focus groups naturally foster within-group interactions, allowing us to gain additional insight into the accuracy of the process map from multiple perspectives. A semistructured guide based on the SEIPS model will elicit feedback on the process map, clarifying its accuracy as a representation of the tasks and procedures performed and identifying system-level barriers and facilitators at each step. The focus group will be audio recorded and is expected to last 90 minutes.

#### Data Analysis

##### Qualitative Analysis

The interview and focus group audio recordings will be transcribed and analyzed using a deductive strategy [70,71] and the SEIPS framework to develop the coding taxonomy, with a focus on identifying barriers and facilitators within each SEIPS category for each step. Our analysis will use triangulation, comparing observed data points from the pCGA visit to those from the patient interviews. The transcripts will be coded independently by 3 researchers, followed by a joint discussion to explore disagreement and achieve consensus. We will use qualitative analysis software NVivo 14 (Lumivero) to catalog coded data.

##### Process Mapping

The aforementioned qualitative analysis and direct observations will be used to adapt the SEIPS-based process map [68]. We will supplement the map with a table, cataloging individual tasks in rows, numbered in reference to the map, with columns listing the persons, environment, and tools and technology, as well as the barriers and facilitators to the completion of each task.

### Phase 2: Participatory Design

#### Study Design

In phase 2, we will apply user-centered participatory design methods to adapt the process map (phase 1) into an implementation package, consisting of a set of implementation strategies, to address barriers and enhance facilitators at each step. Participatory design [72] is a systems engineering method where researchers and participants work together to design the workflow of an intervention. Co-design sessions will be conducted in parallel at a large quaternary academic medical center and an affiliated community-based hospital. There will be 2 groups (design teams) of patients and caregivers and health care professionals per location (a total of 4 groups).

#### Participants

##### Overview

Each design team will consist of 7 participants. As participatory design aims for solution convergence, having an odd number of participants facilitates tie-breaking within groups. The small number of stakeholders in each group, as opposed to 14 in 1 group, allows all stakeholders to provide their input and facilitates critical evaluation.

##### Patients and Caregivers

Older adults are eligible after having completed the pCGA, followed by major abdominal surgery and subsequent recovery. We will approach patients and caregivers at each location (academic hospital and community-based hospital) with the goal to enroll 7 of them for participation. Patients and their caregivers will be approached at the follow-up surgical visit, which is conducted at 14 days postoperatively. Of note, older adults with Alzheimer disease or related dementias will be included if they provide assent and are accompanied by a caregiver who also consents and participates alongside them. We will use purposeful sampling to recruit at least 1 patient or caregiver from underrepresented groups by race, ethnicity, socioeconomic status, and rural or urban backgrounds [69]. We intend to oversample to ensure that perspectives from patients identified as vulnerable are heard.

##### Health Care Professionals

Eligible health care professionals will include surgeons performing major abdominal surgery whose patients may benefit from pCGA and health care professionals who conduct or could potentially conduct elements of the pCGA. For the academic medical center, this will involve the same set of health care professional participants as in phase 1. These include geriatricians, primary care providers, advanced practice providers, social workers, medical assistants, pharmacists, and physical therapists. We will also invite the participation of additional ancillary staff, such as administrative leads, schedulers, and tech support.

#### Data Collection

We will conduct 5 co-design videoconference sessions with each group over 6 months, with a gap of 2 to 3 weeks between each session (Textbox 1; during the periods between the sessions, input from the alternate design team will be synthesized for presentation to the next team and an analysis performed of prior session content). In session 1, we will present the process map generated in phase 1 and a prototype of the current pCGA implementation package. Researchers will facilitate the discussion, maintaining respectful debate among design team members as they identify process changes that will promote the implementation of the pCGA package. The facilitators will focus on solution convergence from session to session. Each design team will participate in sessions independently; however, we will summarize the output from each session and present it to the alternate design team for comment. Each session is expected to last 90 minutes. Sessions will be conducted virtually to promote participation and retention and audio recorded for qualitative analysis. Co-design sessions will be conducted separately for the academic hospital and the community-based hospital.

**Outline of the participatory co-design sessions.**

**Sessions and content for preoperative comprehensive geriatric assessment (pCGA) implementation redesign**

1. Methods introduction, process map review, initial implementation package design
2. Refinement of inputs (eg, referrals)
3. Refinement of pCGA system workflows (eg, telephone calls)
4. Refinement of pCGA output and feedback mechanisms
5. Finalizing implementation package, rollout planning

#### Data Analysis

Qualitative data analysis of the design sessions will be performed using the rapid identification of themes from audio recordings method [73] to facilitate rapid iterative refinement of the implementation package between sessions. After each session, the research team will analyze audio recordings directly without transcription, identifying key themes, an initial codebook, and preliminary analysis of codes. We will synthesize these findings with outputs from the design session, such as sketches, case scenarios, ratings, research team observation notes, and stakeholder notes. This allows early identification of key themes after each session that can then be provided back to stakeholders in the subsequent session. Data analysis will focus on identifying implementation strategies to mitigate perceived barriers or strengthen known facilitators (Textbox 2). We will investigate novel solutions as well as known implementation strategies, such as the 73 discrete strategies delineated in the Expert Recommendations for Implementing Change [74,75].

**Examples of potential implementation strategies based on the systems engineering initiative for patient safety (SEIPS) categories to improve reach and adoption while maintaining fidelity.**

**SEIPS categories and possible strategies**

- People: dedicated referral coordinator (or registered nurse) within surgical clinics
- Organization: clear parameters for referral and script for telephone triage
- Tools: telehealth for remote patients, educational handouts, and order sets
- Tasks: automate recommendations to primary care or referring provider
- Environment: travel vouchers for patients with limited transport

### Phase 3: Pilot Trial of the pCGA Implementation Package

#### Study Design

We will conduct a pretest-posttest pilot study of the implementation package over 10 months, conducted at the clinic level at 2 busy academic medical center surgical subspecialty clinics performing major abdominal surgery (selecting one that does not routinely refer for pCGA in addition to one that does routinely refer). We will collect baseline prepilot data via retrospective chart abstraction from the year before the intervention. After an implementation rollout period of 2 months, postintervention data will be collected over 10 months. Specifically, we will assess outcomes according to the RE-AIM framework [38], using chart abstraction for patient-level data and simple self-reported surveys for the health care professionals. We hypothesize that a user-designed implementation package will improve the reach and adoption of the pCGA compared to the historic baseline.

#### Participants

##### Patients

Older adults presenting to surgical clinics will be screened for eligibility criteria by study staff. Eligible participants are patients aged ≥85 years or those aged ≥65 years with multiple comorbid conditions (>2), diagnosed or suspected cognitive impairment, diagnosed or suspected functional impairment, or poor nutrition (documented weight loss of 10% within a year). The inclusion criterion is eligible older adults planning to undergo elective major abdominal surgery. Exclusion criteria are non-English speakers (because we lack resources for translation) or the absence of a caregiver for those without decisional capacity.

##### Health Care Professionals

Consistent with implementation research, health care professionals using the pCGA implementation package are considered study participants [76]. As participants from among the health care professionals who participated in the co-design will be recruited for the pilot trial, they will be familiar with the intervention. Surgeons referring patients from the 2 clinics (n=12) and health care professionals engaged at the pCGA clinic (n=10) will be asked to participate in the pilot.

##### Recruitment and Enrollment

Clinic staff will approach potential participants to determine interest, and a study team member will then share study details and obtain consent. At baseline, 40 patients per year are seen for pCGA. We expect to enroll 20 patients over the pilot period. All patient data for the pilot will be obtained via chart abstraction. The study will be subject to IRB approval.

##### Intervention

The pCGA implementation package (generated via phase 2) will include a set of implementation strategies [74,75] and an explicit stepwise plan defining the people, tasks, tools or technology, relevant organizational factors, and physical or virtual environment [65] for the pCGA. The implementation package will target systems-level processes to promote pCGA administration before surgery (eg, automating referrals in the electronic medical record, improving patient education materials, or increasing telehealth opportunities).

#### Data Collection and Variables

We will obtain quantitative data from two sources: (1) chart abstraction for patients and (2) self-reported survey instruments for health care professionals.

#### Chart Abstraction

Trained research assistants will prospectively abstract and enter data into a deidentified database on a secure server using a data dictionary. We will conduct random audits for interrater reliability according to best practices for retrospective chart review [77]. Patient-level characteristics will include demographics (age, sex, height, weight, zip code, race, and ethnicity), comorbidities (diabetes, chronic corticosteroid use, cancer diagnosis, congestive heart failure, chronic obstructive pulmonary disease, bleeding disorders or anticoagulant use, and renal insufficiency and dialysis). Clinical data from the pCGA will include polypharmacy (anticholinergic burden score [78] and Beers criteria medications [79]), physical function (Timed Up and Go Test [80], Lawton Independent Activities of Daily Living scale [81], and activities of daily living [82]), cognition (Montreal Cognitive Assessment [83]), mood (Patient Health Questionnaire-9 [84]), nutrition (Mini Nutritional Assessment–Short Form [85] and BMI), multiple chronic conditions (Charlson comorbidity index [86,87] and revised cardiac risk index [88]), documentation of code status, completion of health care power of attorney forms, and recommendations for additional tests or referrals (eg, physical therapy). Clinical data from the surgical hospitalization will include the date of surgery, procedure performed, postoperative complications (pneumonia, myocardial infarction, surgical site infection, sepsis, urinary tract infection, venous thromboembolism, renal failure, readmission, and unplanned reoperation), postoperative delirium, the date of discharge, discharge destination, readmission within 30 days, and death. We will abstract postoperative delirium using a validated method [89-91].

#### Outcomes

The primary outcomes of this pilot trial will be reach and adoption, assessed using the RE-AIM framework [38] (Table 2). Reach refers to the percentage and characteristics of older adults receiving the pCGA intervention, while adoption accounts for those health systems and health care professionals who refer patients for, or deliver, the pCGA [38,92]. Of note, implementation in the RE-AIM framework refers to fidelity, or the extent to which an intervention is delivered as intended. We will expand the implementation domain to include measures of fidelity, as defined by NIH Best Practices [40], as well as feasibility, acceptability, and appropriateness according to the taxonomy of implementation outcomes developed by Proctor et al [93]. The primary effectiveness outcome will be postoperative length of stay, which is an easily measured, continuous outcome variable commonly used in the surgical literature on pCGA [29]. We will collect exploratory measures to inform the selection of effectiveness outcomes for a future implementation-effectiveness trial. These exploratory outcome measures will include (1) a composite of death or postoperative complication events; and (2) data points relevant to the pCGA, including a cognitive outcome (postoperative delirium), functional outcome (discharge destination to home, rehabilitation, or skilled nursing), nutritional outcome (discharge diet, albumin level, and weight at discharge), and polypharmacy (Beers criteria discharge medications [79], anticholinergic burden score [78], and number of opioid pain pills prescribed at discharge).

**Table 2:** The reach, effectiveness, adoption, implementation, and maintenance (RE-AIM) domains and associated outcome measures.

Domain	Definition	Measurement
Reach	The percentage and characteristics of older patients who receive pCGA^a^ before abdominal surgery	Referred ÷ potentially eligible patients Comparison of referred ÷ eligible patients by age, sex, race, ethnicity, BMI, and zip code Target reach: >50% referral of eligible pCGA patients per clinic
Effectiveness	The improvement in clinical outcomes from pCGA receipt	Primary: postoperative length of stay Secondary: 30-day morbidity and mortality (refer to Chart Abstraction section)
Adoption	Surgeon intent, decision, and action to refer eligible patients for pCGA	Percentage of surgeons referring ÷ total surgeons in the pilot clinic Target adoption: >75% of surgeons achieving >50% referrals
Implementation: fidelity	Multifaceted outcome that includes (1) treatment *dose* or amount of intervention delivered, (2) quality or content of intervention delivered by health care professionals, and (3) treatment receipt and enactment by patients (NIH^b^ Best Practices) [40]	(1 and 2) Percentage of completed pCGA components, as abstracted from the medical record at pCGA visit (3) Completion of pCGA recommendations, as abstracted from the medical record at the time of surgery Target fidelity: >90% of pCGA components completed
Implementation: feasibility^c^	Perceived ease of use of pCGA implementation package	Feasibility of intervention measure (4-item survey) [94]
Implementation: acceptability^c^	Satisfaction with the pCGA implementation package	Acceptability of implementation measure (4-item survey) [94]
Implementation: appropriateness^c^	Fit and relevance of pCGA implementation package	Intervention appropriateness measure (4-item survey) [94]
Maintenance	The extent to which pCGA use is sustained over the pilot period	Trends in reach, adoption, and implementation outcomes will be evaluated at 6 months and 12 months

^a^pCGA: preoperative comprehensive geriatric assessment.

^b^NIH: National Institutes of Health.

^c^The feasibility, acceptability, and appropriateness of the pCGA implementation package will be assessed via surveys administered to health care professionals (eg, surgeons, geriatricians, advanced practice providers, nurses, medical assistants, technicians, and staff).

#### Self-Report Surveys

We will administer surveys to health care professionals (surgeons and pCGA staff) at 6 and 12 months into the pilot to assess the feasibility, acceptability, and appropriateness of the implementation package. We will contact health care professionals via email to complete the survey electronically; however, recognizing the demands of clinical schedules, a research assistant will make paper copies of the survey available for completion during clinic hours. We will use a previously validated survey instrument to measure implementation outcomes, specifically the acceptability, appropriateness, and feasibility of the intervention [94]. The self-report survey includes 4 items for each domain (12 items in total) and is estimated to take ≤15 minutes to complete (approximately 5 min/domain). Psychometric assessments of the instrument have demonstrated high validity (Cronbach α values ranging from 0.85 to 0.91) and high reliability (test-retest values ranging from 0.73 to 0.88). The items are scored on a 5-point scale. For each 4-item domain, the score can be calculated by averaging the responses. While cutoff scores have not been established, higher scores indicate greater acceptability, appropriateness, or feasibility.

#### Data Analysis

The aim of the pilot is to maximize reach and adoption while maintaining implementation fidelity before a full-scale implementation-effectiveness hybrid trial is conducted. The limited sample size will restrict our ability to evaluate differences in effectiveness outcomes such as mortality or morbidity; however, more proximal effectiveness estimates will be explored by abstracting short-term clinical outcomes (eg, medication changes at discharge). We will use exploratory measures to establish some preliminary point estimates for event rates and CIs within our sample for the purpose of planning a future study. As such, formal power calculations are not applicable to this pilot study. Recruitment data will inform the calculation of the reach, adoption, and maintenance outcomes. We will explore all RE-AIM outcomes for differences across underrepresented groups [69] (rural vs urban as determined by zip code, racial majority vs minority groups, men vs women vs other gender identities, and Hispanic vs non-Hispanic ethnicity). All data will be entered into Stata software (StataCorp LLC) for analysis. Data will be evaluated for accuracy and missingness. Descriptive univariate and bivariate analyses will be conducted, including chi-square tests for categorical data and 2-tailed *t* tests or Mann-Whitney *U* tests for continuous data as appropriate, with a cutoff of *P*<.05 for statistical significance.

## Results

### Overview

We anticipate that this study will take approximately 60 months to complete, from study start-up procedures to the completion of the pilot trial. The 3 phases will take place between June 2023 and March 2028. We expect the results outlined in the following subsections. The study was funded in April 2023. Phase 1 officially started in May 2023 with the first enrollment June 2023. Data collection were completed in February 2024; however, recruitment was extended specifically for vulnerable populations such as underrepresented minority groups. Phase 1 remains open as of August 2024. This action was made due to low recruitment of underrepresented minority groups in an effort to honor the NIH commitment to diversity, equity, inclusion and accessibility. Phase 2 of the study was initiated in May 2024 and remains ongoing in August 2024.

### Phase 1

The results of this phase will include a detailed process map and the corresponding barriers and facilitators as a starting point for system redesign to improve the implementation of the pCGA, specifically expanding reach while maintaining fidelity.

### Phase 2

The results of the participatory co-design sessions at the academic medical center are expected to converge upon an adaptable pCGA implementation package, ready for feasibility and pilot testing (stage IB of the NIH Stage Model). Stakeholder design sessions at the community-based hospital will serve as a template for user-centered implementation of the pCGA at a future date. The results of co-design at the community-based hospital will provide a starting point for understanding the potential generalizability of the implementation package and the extent to which adaptation may be needed to begin implementation at new sites.

### Phase 3

The pilot trial will test the pCGA implementation package using a pretest-posttest design at 2 surgical clinics enrolling 20 patients in the preimplementation period and 40 in the postimplementation period. Using the RE-AIM framework [38] we will measure reach, effectiveness, adoption, implementation, and maintenance. We aim to improve reach and maintain fidelity. Exploratory analyses will assess differences across traditionally underrepresented groups [69] (rural vs urban, racial majority vs minority groups, men vs women vs other gender identities, and Hispanic vs non-Hispanic ethnicity). We hypothesize that reach will increase while maintaining fidelity in the postimplementation phase of the pilot.

### Dissemination

The findings from each phase of the study will be disseminated following best practices throughout the conduct of this research. We will publish peer-reviewed manuscripts and present findings at professional conferences for each phase of the study. The results will be posted to ClinicalTrials.gov within 12 months of study completion.

## Discussion

### Anticipated Principal Findings

This study will apply both implementation science and human factors engineering methods aiming to generate the following main findings for each phase of the study: (1) detailed process map for the pCGA, (2) a co-designed implementation package to improve the use of the pCGA among older patients having major abdominal surgery, and (3) preliminary data to support a future implementation-effectiveness trial. In addition to the aforementioned expected output, the study will allow us to explore our hypothesis that systems engineering methods of process mapping and co-design can successfully be applied to the pCGA in surgical care (aims 1 and 2) and that a rigorous user-centered pCGA implementation package will improve surgical care processes (reach and adoption, which are the primary implementation outcomes) and patient outcomes (length of stay, which is the primary effectiveness outcome) for older adults undergoing major abdominal surgery.

### Comparison to Prior Work

The findings will be interpreted in the context of prior literature. There are few studies addressing pCGA implementation challenges. Implementation science can provide insight into the heterogeneity of the literature on the pCGA. Poor implementation of the pCGA contributes to variable effectiveness among older adults undergoing abdominal surgery. Two randomized controlled trials involving patients undergoing abdominal surgery have been conducted; however, both were underpowered, with significant variability in the fidelity to, and the implementation of, the pCGA [95,96]. Hempenius et al [95] randomized a heterogeneous group of 260 patients with cancer undergoing minor, intermediate, or major operations (eg, abdominal surgery) to usual care or an in-hospital “geriatric liaison” service conducting pCGA tests 24 hours before the operation. The authors found a 34% relative reduction in postoperative delirium, but it was not significant due to an underpowered sample size and a lower-than-expected rate of delirium [95]. The effect of the intervention was likely reduced because the “liaison” model leaves inadequate time to reduce risks, and minor procedures carry very low complication rates with lower postoperative delirium rates. Ommundsen et al [96] randomized a population of patients with frailty with colorectal cancer to receive outpatient pCGA versus usual care, finding no significant difference in the primary outcome of severe surgical complications [96]. This study was limited by the lack of a multidisciplinary team supporting the intervention, inadequate preoperative time to implement pCGA recommendations, unclear patient adherence to recommendations, and an inability to reach the necessary sample size. This resulted in an underpowered study and a weak intervention with poor fidelity.

### Strengths

By applying an implementation focus, this study will define system-level strategies to maintain fidelity while improving implementation, thereby elevating the quality of evidence in examining the effectiveness of the pCGA. We expect that this study will facilitate a paradigm shift toward rigorous implementation-effectiveness [97] work to enhance perioperative care for older patients undergoing surgery. Our line of research is innovative because it aims to refine the implementation of an existing intervention, engaging an affiliate community-based hospital in user-centered design, to expand the reach of the pCGA to patients in a variety of settings. Our methods are also innovative, applying human factors engineering methods and the SEIPS model to a new field: the use of the pCGA among older patients having major abdominal surgery.

### Limitations

We anticipate that there will be limitations and potential challenges in conducting this study. Recruitment and retention will be addressed by incentivizing participation (gift cards) and recruiting at least 10% more participants than required. As user-centered design sessions will require continuity, we will conduct team-building exercises to promote investment in the process and provide sufficiently generous incentives to retain participants. Smaller design teams are often more effective, and we will accept 2 dropouts per group. Patient and caregiver access to technology may limit participation in video-based assessments in phases 1 and 2; however, we believe that this challenge can be overcome because these methods have successfully been applied over the course of the COVID-19 pandemic. Alternative strategies such as in-person attendance of interviews and design sessions also risk potential limitations or exclusion of those with poor access to transportation. We recognize that the process map within the academic medical center may not be generalizable to the community-based hospital. It is possible that the pCGA implementation package cannot be successfully adapted to the community-based hospital. In this case, user-centered design will allow us to explore the pCGA components that will be most valuable to the health care professionals and patients at the community-based hospital. This may require a shift toward intervention adaption, evaluating a pared-down, community-specific pCGA intervention. The implementation package and pilot test in phase 3 will focus on system-level changes; however, we anticipate identifying individual-level barriers and facilitators to implementation (primary care provider follow-through or patient barriers to performing exercises). The role of individual behavior in fidelity to the intervention cannot be ignored and may serve as an area for future research. We plan to track these findings; however, interventions to alter individual behavior are beyond the scope of this system-level investigation.

### Future Directions

This study will produce preliminary data on which to build a future implementation-effectiveness trial. Upon successful adaptation of the implementation package, we anticipate performing a step-wedge randomized controlled trial to implement the pCGA at multiple sites. The anticipated type II hybrid implementation-effectiveness trial will equally weigh implementation and effectiveness outcomes. It will track both short-term (30-day) and longer-term (90- and 120-day) outcomes, including morbidity and mortality, and, as noted in the *Outcomes* section, will include outcomes across the pCGA domains (cognition, function, nutrition, polypharmacy, mood, and quality of life). If successful, the adaptable pCGA implementation package will provide an actionable strategy for hospitals seeking to improve surgical care for older adults. With an effective intervention and a well-tested implementation strategy, future work will focus on scalability and dissemination. By engaging an affiliate community-based hospital in user-centered design, this research will develop implementation strategies by which to expand the reach of the pCGA. We will explore the differential receipt and experience of the pCGA across diverse populations, consistent with the NIH commitment [98] to diversity, equity, and inclusion, including the study of health equity. Throughout the study, we will engage and elicit the voices of patients and caregivers traditionally underrepresented in medical research [69] according to race, ethnicity, socioeconomic status, and rural or urban backgrounds. In addition to hybrid implementation-effectiveness trials, future directions will focus on adaptation to new populations, dissemination strategies, and the scalability of the intervention.

### Dissemination Plans

We anticipate that surgeons and hospitals participating in statewide quality improvement collaboratives, such as the Surgical Collaborative of Wisconsin, may be interested in interventions that aim to improve surgical care for older adults. Larger national initiatives, such as the American College of Surgeons Geriatric Surgery Verification Program [99], require the performance of a pCGA. If successful, the pCGA implementation package may serve as a valuable resource and provide an actionable strategy for hospitals in these or similar programs. Our ability to provide an effective intervention with a well-tested implementation strategy will open the possibility of regional or national dissemination.

### Conclusions

Upon completion, the study will produce a comprehensive and rigorously evaluated pCGA implementation package as well as feasibility data to support a multisite hybrid implementation-effectiveness clinical trial. This study will advance the field, improving the quality of implementation data on the pCGA, providing replicable implementation strategies for the pCGA, and integrating human factors methods into perioperative science. It will further our overall goal to improve surgical care for older adults through transdisciplinary research that promotes the implementation and dissemination of effective, evidence-based, patient-oriented interventions.

## Abbreviations

IRB: institutional review board
NIH: National Institutes of Health
pCGA: preoperative comprehensive geriatric assessment
RE-AIM: reach, effectiveness, adoption, implementation, and maintenance
SEIPS: systems engineering initiative for patient safety

## Data Availability

The data sets generated and analyzed during this study will be available in deidentified form after study completion from the corresponding author on reasonable request.
